# The efficacy and safety of different does of intravenous tranexamic acid on blood loss in fresh foot and ankle fractures: a prospective, randomized controlled study

**DOI:** 10.1186/s12891-024-07410-4

**Published:** 2024-04-09

**Authors:** Gang Tan, Jia Li, Jing Xu, Yongzhan Zhu, Hui Zhang

**Affiliations:** 1 Department of Orthopaedics, West China Hospital of Sichuan University, No.37 of Guoxue lane, Wuhou District, Chengdu, Sichuan 610041 China; 2 Department of Orthopaedics, West China School of Public Health and West China Fourth Hospital, Sichuan University, Chengdu, Sichuan China; 3 Operating Room, West China School of Nursing, West China Hospital, Sichuan University, Chengdu, 610041 China; 4 Foshan Hospital of Traditional Chinese Medicine, Foshan, Guangdong China

**Keywords:** Tranexamic acid, Efficacy and safety, Different does, Blood loss, Fresh foot and ankle fractures

## Abstract

**Background:**

There are a few studies on the effectiveness and safety of intravenous administration of tranexamic acid(TXA) in patients who underwent foot and ankle surgery, especially for preoperative hidden blood loss in patients with freshfoot and ankle fractures. Thus, the aim of this study was to investigate whether intravenous administration of different doses of TXA can effectively reduce perioperative blood loss and blood loss before surgery and to determine its safety.

**Methods:**

A total of 150 patients with fresh closed foot and ankle fractures from July 2021 to July 2023 were randomly divided into a control group (placebo controlled [PC]), standard-dose group (low-dose group [LD], 1 g/24 h; medium-dose group [MD], 2 g/24 h), and high-dose group (HD, 3 g/24 h; ultrahigh-dose group [UD], 4 g/24 h). After admission, all patients completed hematological examinations as soon as possible and at multiple other time points postsurgery.

**Results:**

There was a significant difference in the incidence of hidden blood loss before the operation between the TXA group and the control group, and the effect was greater in the overdose groups than in the standard-dose groups. There were significant differences in surgical blood loss (intraoperative and postoperative), postoperative HGB changes, and hidden blood loss among the groups. The TXA groups showed a significant decrease in blood loss compared to that of the control group, and the overdose groups had a more significant effect than the standard-dose groups. A total of 9 patients in the control group had early wound infection or poor healing, while only 1 patient in the other groups had this complication, and the difference among the groups was significant. No patients in any group suffered from late deep wound infection, cardiovascular or cerebrovascular events or symptomatic VTE.

**Conclusion:**

This is the first study on whether TXA can reduce preoperative hidden blood loss in patients with freshfoot and ankle fractures. In our study, on the one hand, intravenous application of TXA after foot and ankle fractures as soon as possible can reduce preoperative blood loss and postoperative blood loss. On the other hand, TXA can also lower wound complications, and over-doses of TXA are more effective than standard doses. Moreover, overdoses of TXA do not increase the incidence of DVT.

## Introduction

Foot and ankle fractures are common fractures in the clinic and include ankle joint fractures, pilon fractures, calcaneal fractures, and lower tibial fractures; these fractures are often caused by high-energy injuries [1]. Due to poor soft tissue quality, incision complications, such as poor wound healing, wound infection, or joint infection, often occur after surgery [2, 3]. Transexamic acid (TXA) is an artificially synthesized lysine analog that was first reported by the Okamoto couple [4]. Although TXA has been widely used in orthopedics [5–7] and in a few studies on the effectiveness and safety of intravenous administration of TXA in patients with foot and ankle surgery [8–10], multiple randomized controlled clinical trials have shown that overdose of TXA does not increase the incidence of thrombosis events in other diseases [11–13]. However, whether TXA can lower preoperative hidden blood loss in fresh foot and ankle fractures is unknown, and there is a lack of direct evidence to prove whether a high dose of TXA can effectively reduce the incidence of complications but not increase DVT in foot and ankle fractures. The purpose of our research was to determine whether TXA can effectively reduce perioperative blood loss, especially preoperative blood loss, and whether a high dose of TXA can efficiently reduce incision complications without increasing the risk of symptomatic DVT in fresh foot and ankle fractures.

## Methods

### Participants

Patients who suffered fresh foot and ankle fractures and who were admitted to our hospital between July 2021 and July 2023 were included and randomly divided into a control group (placebo controlled [PC]), standard-dose group (low-dose group [LD], 1 g/24 h; medium-dose group [MD], 2 g/24 h), and high-dose group (HD, 3 g/24 h; and URD, 4 g/24 h).

### Inclusion criteria

(i) had closed freshfoot and ankle fractures and a duration from injury to admission of less than 6 h; (ii) were aged ≥ 18 years; (iii) had no history of TXA allergy; (iv) were able to tolerate anesthesia and surgery; and (v) had no history of severe liver, kidney, heart, lung, or blood diseases.

### Exclusion criteria

(i) history of epilepsy; (ii) contraindications to TXA, including renal failure (serum creatinine > 200 mmol/L, creatinine clearance < 50 ml/min, or dialysis), current use of anticoagulants, severe coronary artery disease or disseminated intravascular coagulation; (iii) past history of thrombosis (myocardial infarction, cerebral infarction, deep vein thrombosis, pulmonary embolism); (iv) pregnancy; (v) massive upper urinary tract bleeding (caused by thrombosis) risk of ureteral obstruction); (vi) bleeding disorders/coagulation disorders.

### Outcome measurement

#### Primary outcome

The demographic data included hemoglobin (HGB), C-reactive protein (CRP), transfusion rate, estimated intraoperative blood loss (IBL), hidden blood loss (HBL) and total blood loss (TBL) data.

#### Secondary outcome

Infections, thromboembolic events, nonunion rate at 6 months.

#### Hematology examination

On the day of admission (D0), before TXA administration; on the 1st (D1), 2nd (D2), and 3rd (D3) days after TXA administration; and the day before surgery (Preop), 1st (postoperative 1d), 3rd (postoperative 3d), and 7th days (postoperative 7d) after surgery, routine blood examination, coagulation, D-dimer, and CRP were performed.

#### DVT

Deep vein ultrasound examination of both lower limbs was performed 24 h after admission; 3, 5, and 7 days after injury; and 1 day before surgery to assess the presence of deep vein thrombosis(DVT). If asymmetric swelling of both lower limbs occurred during hospitalization, deep vein ultrasound examination was performed on the lower limbs at any time.

### Blood loss

The methods used to calculate surgical estimated IBL, visible blood loss (VBL), HBL and TBL were previously described [14, 15].

Preoperative HCT was defined as the HCT within 1 day before surgery. However, only one article has studied the impact of TXA on preoperative blood loss in elderly patients with hip fractures; this study included HCT on the day of admission and the third day after admission following the Gross formula for calculation [16]. Therefore, we also selected the HCT on the day of admission and the third day after admission.

The estimated IBL was calculated as the volume of liquid in the negative pressure aspirator-the volume of flushing saline + the net weight gain of the gauze. The preoperative weight of the dry gauze was precisely specified. Thereafter, the circulating nurse weighed the gauze used with an electronic scale after the surgery. Finally, we obtained the net weight gain of the gauze.

The VBL is the sum of the estimated IBL and postoperative drainage volume.

The HBL was calculated as follows. First, the patient’s blood volume (PBV) was calculated according to the formula:

PBV (mL) = [k1×height (m) 3 + k2 ×weight (kg) + k3] ×1000, where k1 = 0.3669, k2 = 0.03219, and k3 = 0.6041 for male patients, whereas k1 = 0.3561, k2 = 0.03308, and k3 = 0.1833 for female patients. Next, the Gross equation was used to calculate HBL based on HCT:

HBL = PBV×2×(preoperative HCT - HCT at 72 h postoperative)/(preoperative HCT + HCT at 72 h postoperative) + autologous blood transfusion volume + allogeneic blood transfusion volume - VBL.

$$TBL = VBL + HBL$$

### Statistical analysis

All statistical analyses were completed using SPSS 25.0 software (IBM, Chicago, IL, USA). Normally distributed continuous variables are expressed as the mean ± standard deviation, and nonnormally distributed continuous variables are expressed as the median and interquartile range. Continuous variables were compared using one-way analysis of variance. When significant differences were detected, the least significant difference (LSD) method, was used to compare the groups. Categorical variables were compared using the chi-square test and Fisher’s exact probability method when necessary. A p value < 0.05 was considered to indicate statistical significance.

## Results

### General information

**Fig. 1:**
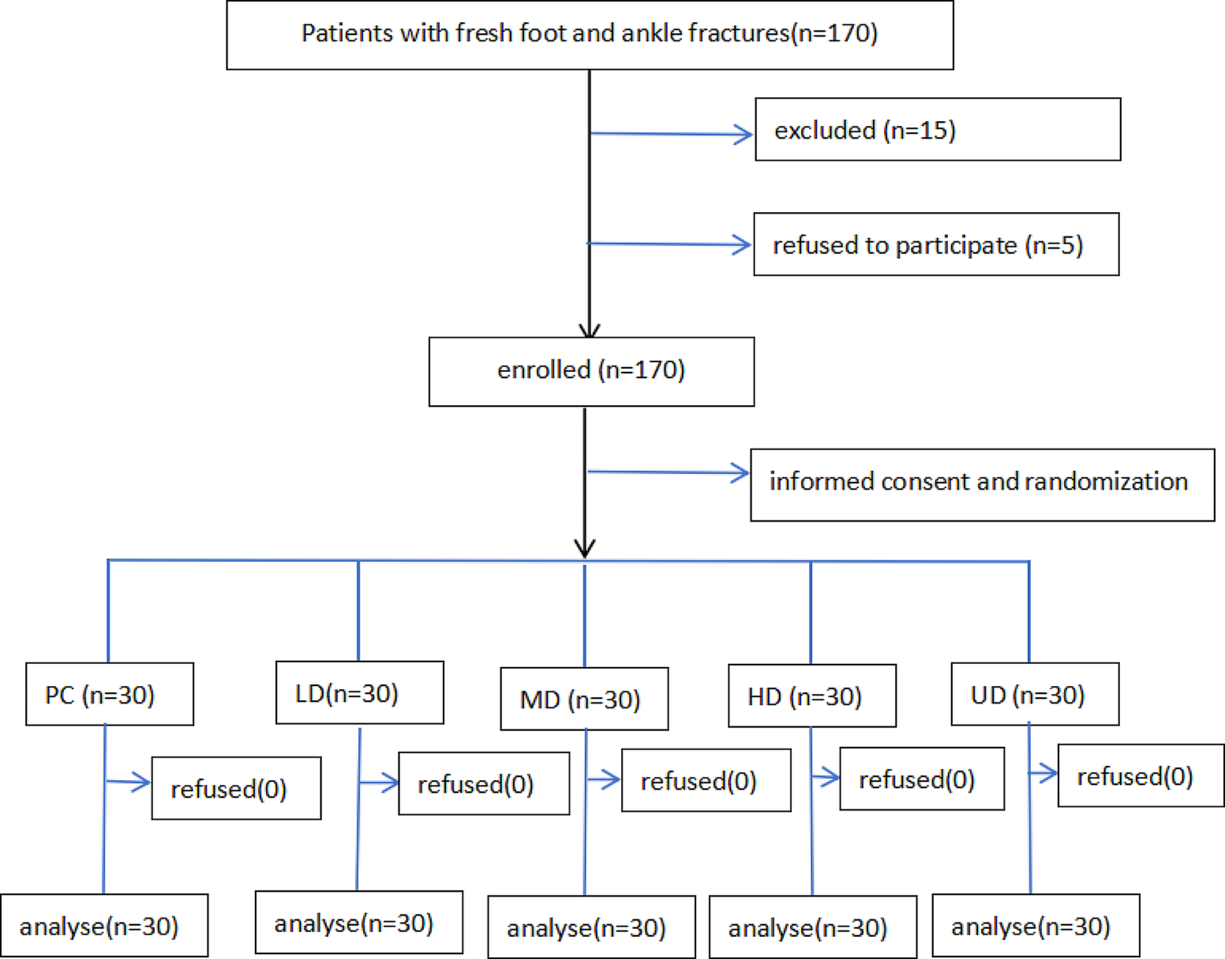
Patient selection and exclusion flow chart. Abbreviation: PC: control group; LD: low-dose group; MD: medium-dose group; HD: high-dose group; UD: ultra-high-dose group

A total of 150 patients were ultimately included in this study. The general demographic information of these patients is shown in Table 1; Fig. 1, and there were no significant differences between the groups.

**Table 1:** general information of 150 patients with fresh foot and ankle fractures

Variable	PC(30)	LD(30)	MD(30)	HD(30)	UD(30)	*P*
Age (years)	46.7 ± 12.99	44.03 ± 12.23	51.1 ± 14.32	43.6 ± 11.11	49.37 ± 18.75	0.182
Sex (male/female)	17/13	15/15	18/12	15/15	19/11	0.783
BMI (kg/m^2^)	23.96 ± 1.82	23.77 ± 3.31	23.89 ± 1.31	23.45 ± 2.87	23.42 ± 1.97	0.855
diagnosis (*n*, %)						
Ankle and Pilon Fractures	20(66.67%)	21(70.00%)	19(63.33%)	18(60.00%)	22(73.33%)	0.827
Calcaneal fracture	10(27.59%)	9(23.33%)	11(24.14%)	12(23.33%)	8(23.33%)	
Comorbidities (*n*, %)						
Diabetes	3(20.69%)	3(16.67%)	4(13.80)	5(16.67%)	4(20.00%)	0.935
Smoking	12(20.69%)	11(23.33%)	10(24.14%)	12(20.00%)	13(27.59%)	0.953
Alcoholism	7(24.14%)	8(26.67%)	8(27.59%)	8(27.59%)	6(20.00%)	0.976
Gout	1(3.45%)	1(6.67%)	1(6.89%)	1(3.45%)	2(6.89%)	0.958
Glucocorticoid usage	1(6.89%)	1(3.33%)	1(3.45%)	0(6.89%)	1(3.45%)	0.914

Abbreviation: COPD, chronic obstructive pulmonary disease;

### Blood loss before the operation

**Fig. 2:**
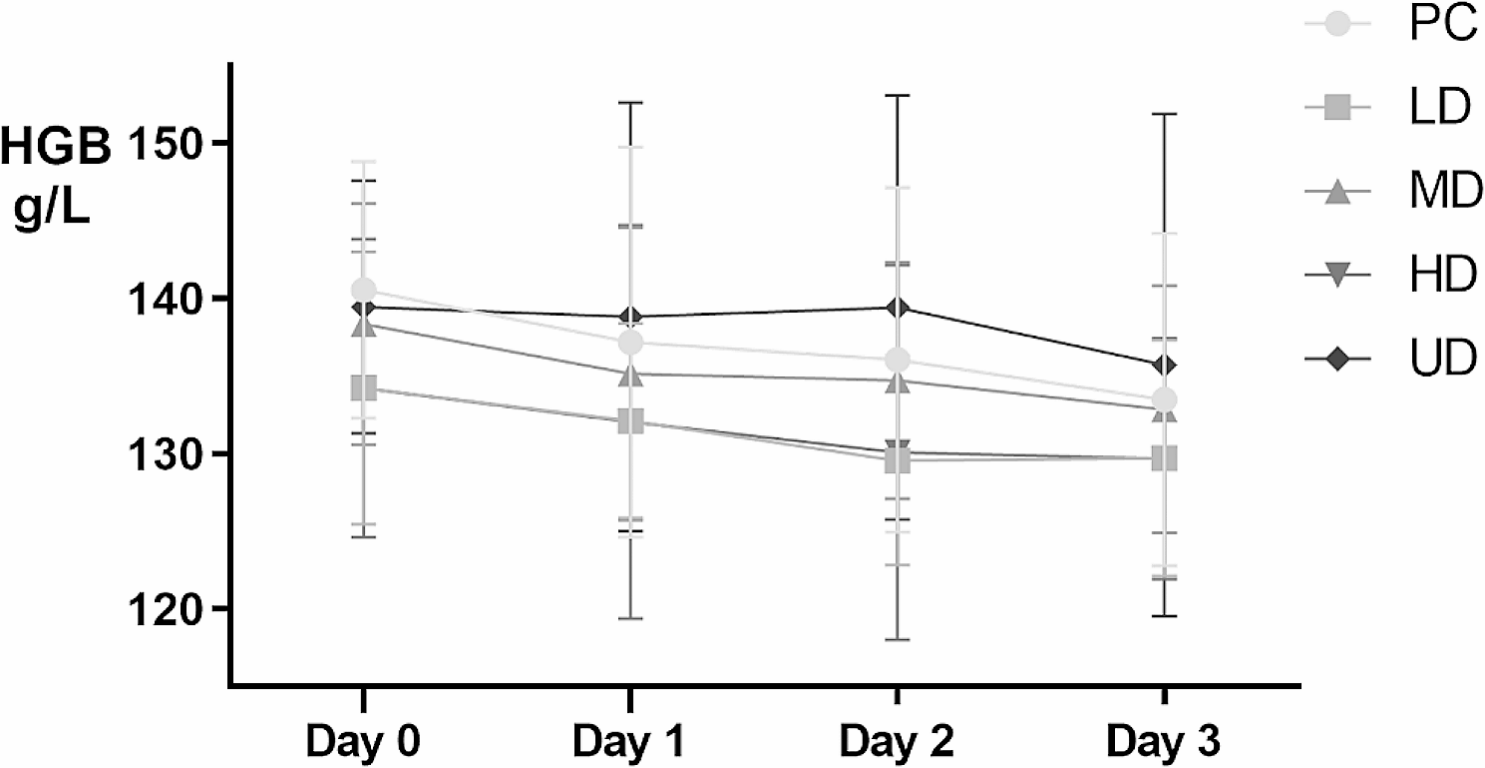
The HGB variation of 150 patients with fresh foot and ankle fractures before operation

**Fig. 3:**
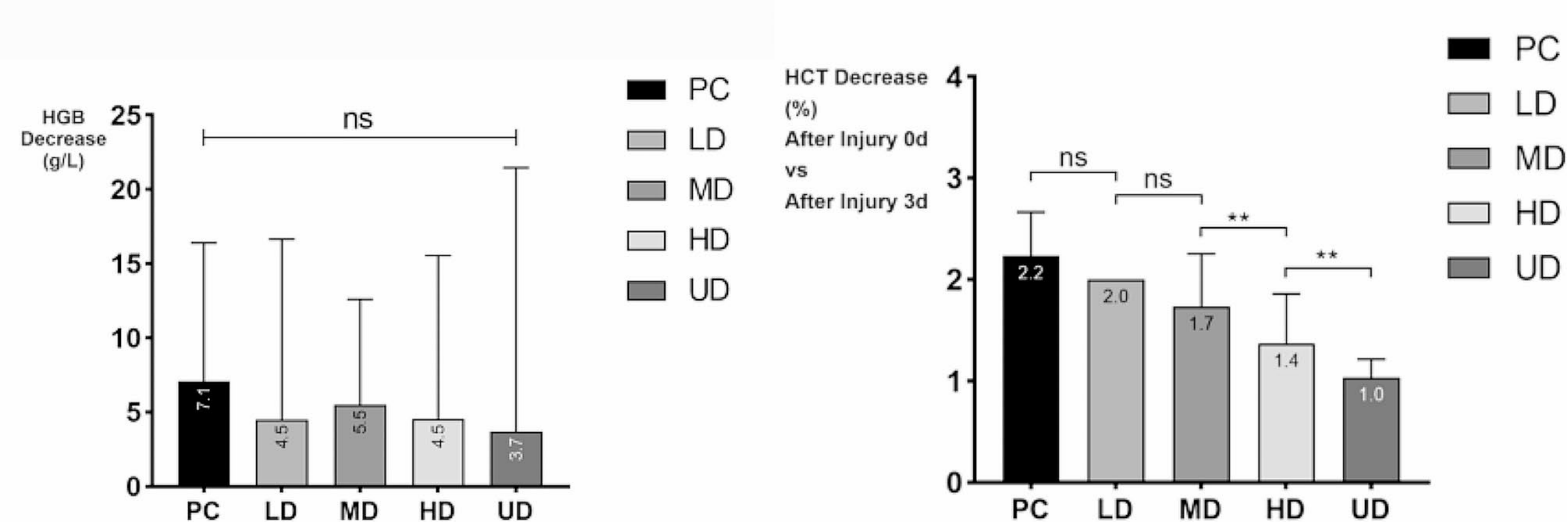
The HGB and HCT variation of 150 patients with fresh foot and ankle fractures before operation(Day 0-Day 3)

**Fig. 4:**
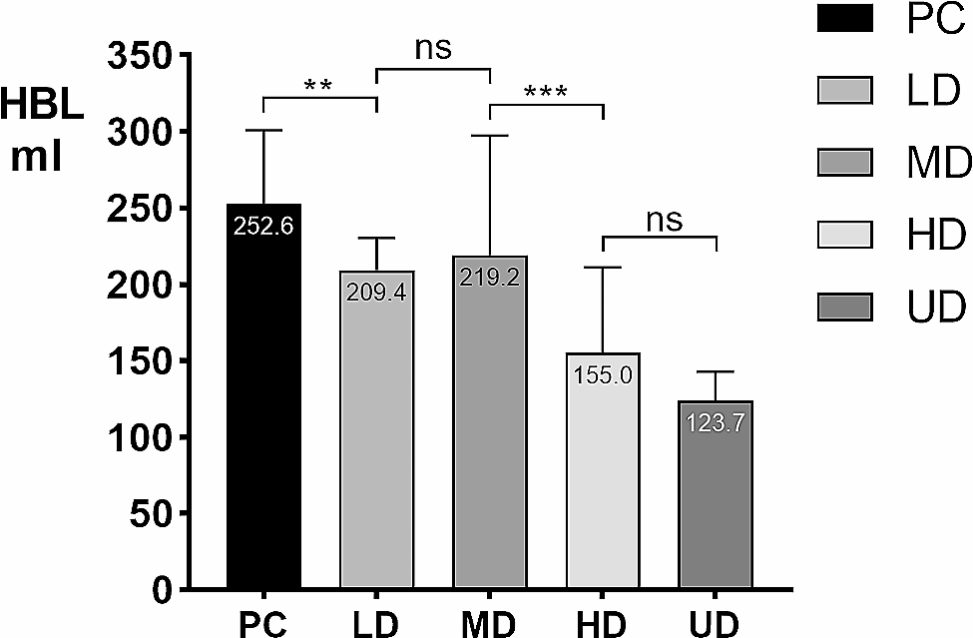
The HBL variation of 150 patients with fresh foot and ankle fractures before operation

There were no significant differences in HGB or HGB among the groups on the 3rd day after admission, but there were significant differences in HGB and HCT on the day of admission, as well as in HCT, HCT variation and HBL on the third day after admission. On the day of admission (Day 0), the first day (Day 1), the second day (Day 2), and the third day (Day 3) after admission, the HGB level in each group showed a downward trend. The difference in HGB between Day 0 and Day 3 was not significant among the five groups. For HCT, there was a significant difference in the difference in HCT between Day 0 and Day 3 among the five groups, with a higher TXA dose resulting in a smaller reduction in HCT. For HBL, there was a significant difference in preoperative HBL among the groups. The TXA group had less preoperative HBL than the control group, while the HD and UD groups had less preoperative HBL than the LD and MD groups. The details are shown in Table 2; Figs. 2, 3 and 4.

**Table 2:** preoperative blood loss in 150 patients with fresh foot and ankle fractures

	PC(30)	LD(30)	MD(30)	HD(30)	UD(30)	P*
Day 0 HGB (g/L)	140.5 ± 8.3	134.2 ± 8.8	138.3 ± 7.8	134.2 ± 9.6	139.4 ± 8.2	**0.008**
Day 1HGB (g/L)	133.5 ± 10.7	129.7 ± 7.6	132.8 ± 8.0	129.7 ± 7.8	135.7 ± 16.2	0.132
HGB variation	7.1 ± 9.3	4.5 ± 12.2	5.5 ± 7.1	4.5 ± 11.0	3.7 ± 17.8	0.847
Day 0 HCT (%)	41.4 ± 1.8	39.3 ± 2.5	36.4 ± 5.2	38.0 ± 4.0	39.1 ± 6.2	**<0.001**
Day 3 HCT (%)	39.2 ± 1.5	37.3 ± 2.5	34.7 ± 5.1	36.6 ± 3.8	38.1 ± 6.1	**<0.001**
HCT variation (%)	2.2 ± 0.4	2.0 ± 0.0	1.7 ± 0.5	1.4 ± 0.5	1.0 ± 0.2	**<0.001**
HBL (ml)	252.6 ± 48.1	209.4 ± 20.9	219.2 ± 78.3	155.0 ± 56.0	123.7 ± 19.0	**<0.001**

Abbreviation: HGB, hemoglobin; HCT, hematocrit *:One-Way ANOVA

### Surgical blood loss

There were significant differences in HGB and HCT, HGB and HCT variation, HBL, IBL, and TBL after surgery. There were no significant differences in HGB or HCT among the five groups one day before surgery, there were no postoperative blood transfusion events in any of the TXA groups, and only one patient in the PC group needed blood transfusion.

**Fig. 5:**
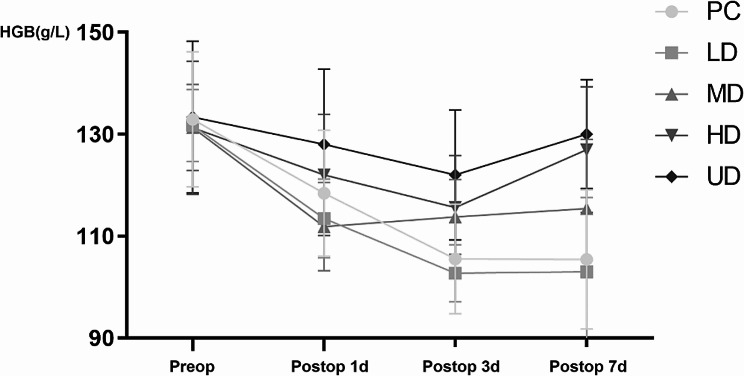
The HGB variation of 150 patients with fresh foot and ankle fractures after operation

**Fig. 6:**
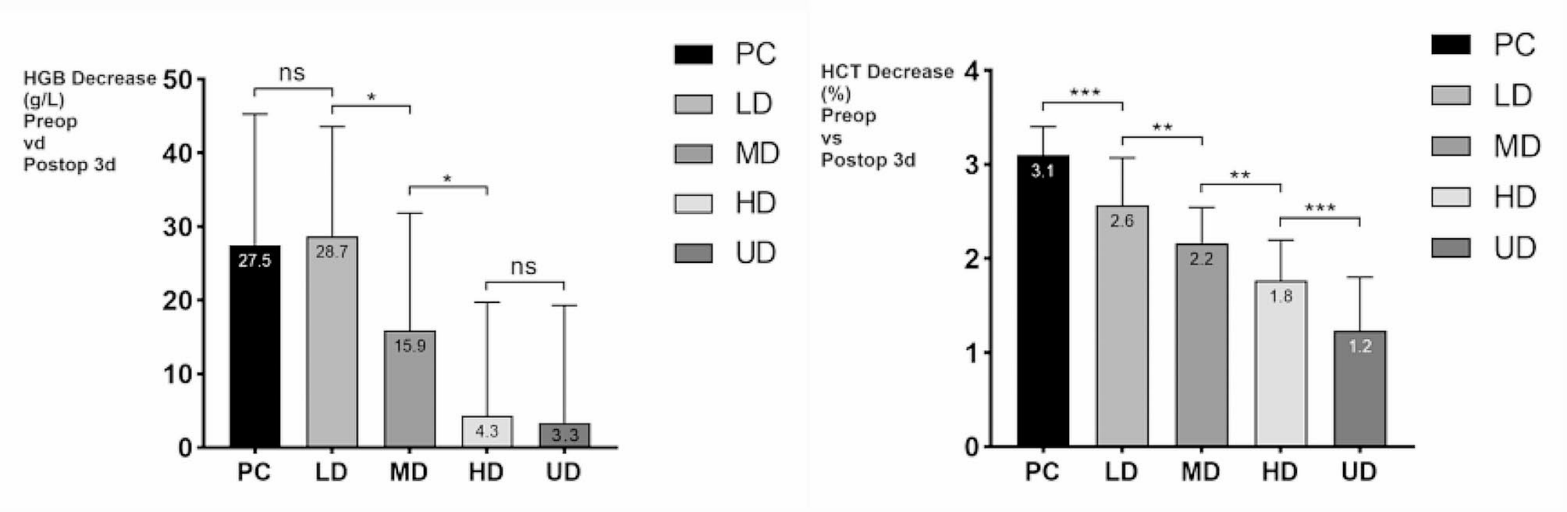
The HGB and HCT variation of 150 patients with fresh foot and ankle fractures (Preop-Postop 3d)

**Fig. 7:**
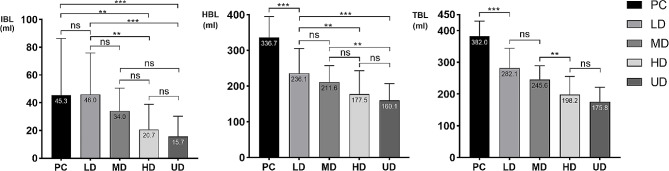
The surgical IBL, HBL, TBL of 150 patients with fresh foot and ankle fractures

On the day before surgery (Preop), the first day (Postop 1d), the third day (Postop 3d) and the seventh day (Postop 7d) after surgery, the HGB levels in each group first decreased and subsequently increased. Moreover, the rate of decrease of HGB in the TXA groups was slower than that in the control group, and the rate of decrease in the HD and UD groups was slower than that in the LD and MD groups. In addition, the difference in HGB between Preop and Postop was smaller in the MD, HD, and UD groups than in the PC and LD groups, and the HGB variation in the HD and UD groups was greater. There were significant differences in HBL and TBL among the groups, with fewer HBL and TBL in the TXA group than in the control group and fewer HBL and TBL in the HD and UD groups than in the LD and MD groups. The details are shown in Table 3; Figs. 5, 6 and 7.

**Table 3:** surgical blood loss in 150 patients with fresh foot and ankle fractures

	PC(30)	LD(30)	MD(30)	HD(30)	UD(30)	P*
Preop HGB (g/L)	132.9 ± 13.3	131.7 ± 7.1	131.3 ± 8.5	131.3 ± 13.1	133.4 ± 14.9	0.937
Postop 3d HGB (g/L)	105.4 ± 13.7	103.0 ± 14.5	115.4 ± 13.6	127.0 ± 12.4	130.0 ± 10.7	**<0.001**
HGB variation	27.5 ± 17.9	28.7 ± 14.9	15.9 ± 16.0	4.3 ± 15.4	3.3 ± 16.0	**<0.001**
Preop HGB HCT (%)	38.9 ± 4.7	37.7 ± 2.7	40.6 ± 5.7	39.2 ± 3.2	39.8 ± 5.6	0.179
Postop 3d HCT (%)	35.8 ± 4.5	35.2 ± 2.8	38.4 ± 5.5	37.5 ± 3.3	38.5 ± 5.7	**0.011**
HCT variation	3.1 ± 0.3	2.6 ± 0.5	2.2 ± 0.4	1.8 ± 0.4	1.2 ± 0.6	**<0.001**
HGB (ml)	336.7 ± 58.3	236.1 ± 69.6	211.6 ± 45.9	177.5 ± 65.5	160.1 ± 47.2	**<0.001**
IBL(ml)	45.3 ± 41.0	46.0 ± 29.9	34.0 ± 16.5	20.7 ± 18.2	15.7 ± 14.6	**<0.001**
TBL (ml)	382.0 ± 48.0	282.1 ± 62.2	245.6 ± 43.6	198.2 ± 57.5	175.8 ± 45.5	**<0.001**
Blood transfusion(n)	1(3.33%)	0(0%)	0(0%)	0(0%)	0(0%)	1.000

Abbreviation: HGB, hemoglobin; HCT, hematocrit *:One-Way ANOVA

### CRP

**Fig. 8:**
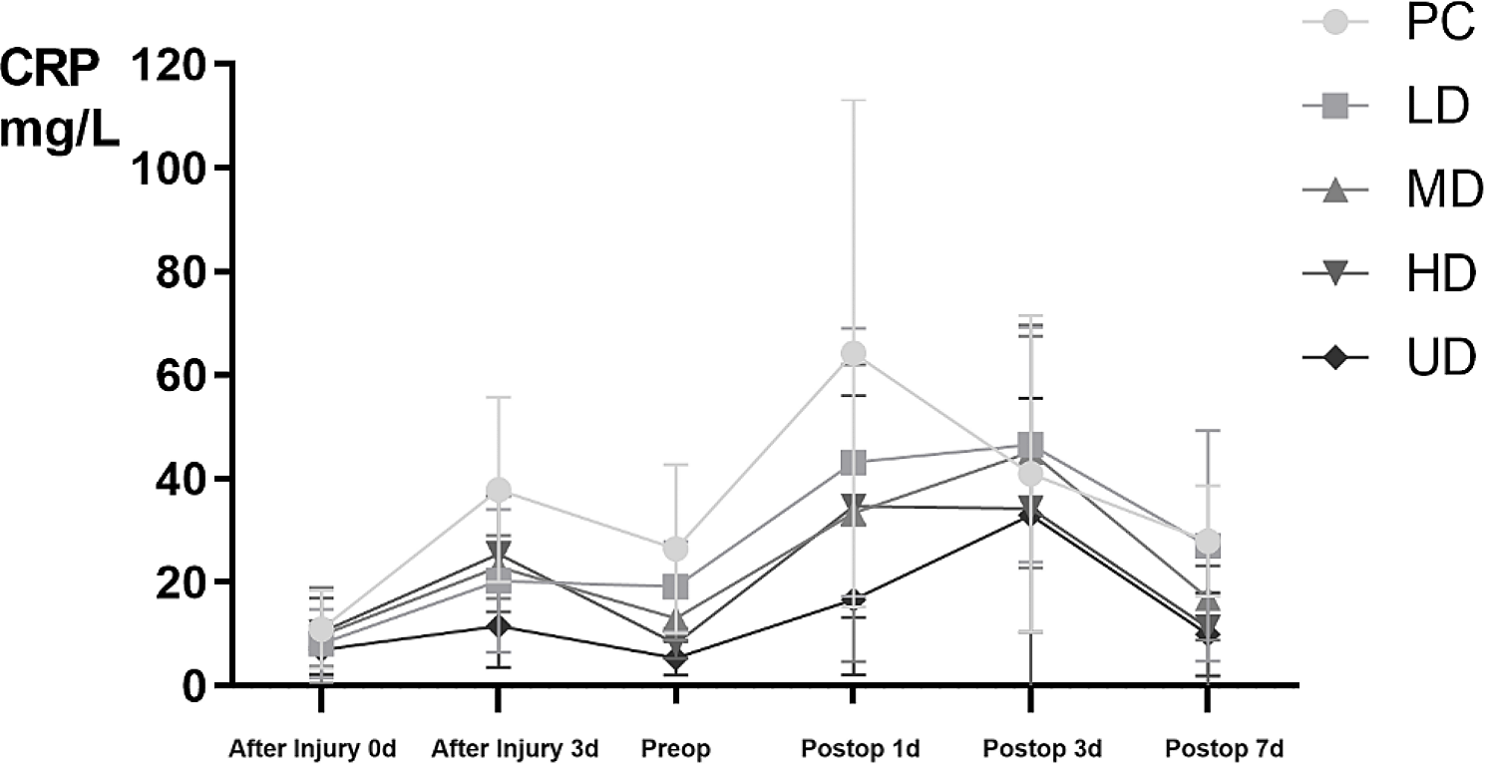
The CRP level of 150 patients with fresh foot and ankle fractures

On the day of admission (after injury 0 d), the 3rd day after admission (after injury 3 d), one day before surgery (Preop), the 1st day (Postop 1 d), the 3rd day (Postop 3 d), and the 7th day (Postop 7 d) after surgery, the CRP levels in all groups first increased and subsequently decreased; however, the rates of increase and decrease in the tranexamic acid groups were slower than those in the control group, and the rates in the HD and UD groups were slower than those in the LD and MD groups. The details are shown in Fig. 8.

### Coagulation and fibrinolysis

**Fig. 9:**
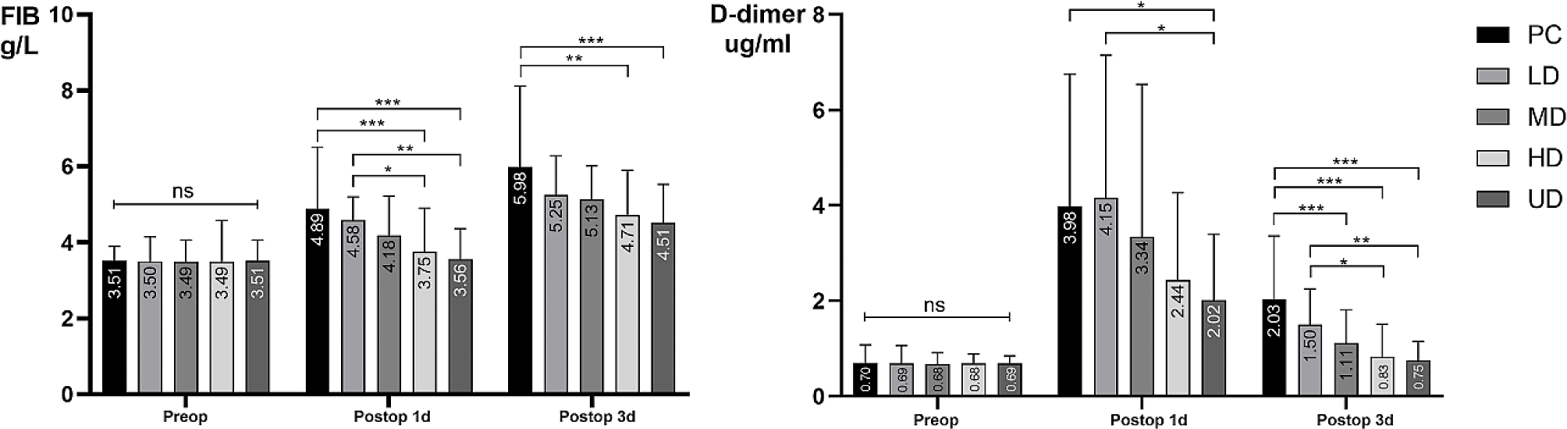
Change trend of FIB/D-dimer in patients with fresh foot and ankle fractures

In this study, there were no significant differences in preoperative or postoperative PT or INR among the groups, indicating that TXA did not affect coagulation. FIB and D-dimer levels showed a downward trend after TXA administration, and the greater the dose of TXA was, the more obvious the downward trend was. The details are shown in Table 4; Fig. 9.

**Table 4:** Preoperative and postoperative coagulation and fibrinolysis indicators in trauma patients

Variable	PC(30)	LD(30)	MD(30)	HD(30)	UD(30)	*P* *
**PT** (s)						
d0	11.77 ± 1.50	11.34 ± 0.48	11.24 ± 0.54	11.50 ± 0.60	11.77 ± 0.85	0.601
d3	11.87 ± 1.86	11.52 ± 0.22	11.79 ± 0.18	11.59 ± 0.24	11.66 ± 0.32	0.513
D1	12.53 ± 2.06	11.77 ± 0.20	12.41 ± 0.18	12.18 ± 0.57	12.13 ± 0.43	0.072
D3	11.66 ± 2.38	11.62 ± 0.20	11.66 ± 0.58	11.54 ± 0.65	11.47 ± 0.21	0.965
**INR**						
d0	0.98 ± 0.06	0.98 ± 0.04	0.96 ± 0.03	0.99 ± 0.05	0.99 ± 0.06	0.617
d3	1.00 ± 0.31	0.99 ± 0.02	1.01 ± 0.01	0.98 ± 0.02	0.99 ± 0.03	0.948
D1	1.04 ± 0.30	1.04 ± 0.03	1.07 ± 0.01	1.05 ± 0.05	1.05 ± 0.03	0.892
D3	0.97 ± 0.14	0.99 ± 0.02	0.99 ± 0.05	0.99 ± 0.06	0.97 ± 0.02	0.441
**FIB (g/L)**						
d0	3.4 ± 0.45	3.25 ± 0.61	3.36 ± 0.66	3.28 ± 0.62	3.29 ± 1.01	0.923
d3	4.42 ± 1.19	4.14 ± 0.70	3.75 ± 0.42	3.41 ± 1.42	3.41 ± 0.56	**<0.001**
D0	3.51 ± 0.37	3.50 ± 0.64	3.49 ± 0.57	3.49 ± 1.08	3.51 ± 0.55	0.982
D1	4.89 ± 1.60	4.58 ± 0.61	4.18 ± 1.03	3.75 ± 1.15	3.56 ± 0.80	**<0.001**
D3	5.98 ± 2.13	5.25 ± 1.02	5.13 ± 0.88	4.71 ± 1.19	4.51 ± 1.01	**<0.001**
**D-dimer (ug/ml)**						
d0	0.43 ± 0.19	0.48 ± 0.26	0.46 ± 0.14	0.37 ± 0.21	0.42 ± 0.19	0.293
d3	1.13 ± 0.57	1.03 ± 0.79	0.94 ± 0.28	0.99 ± 0.25	0.72 ± 0.26	**<0.001**
D0	0.70 ± 0.39	0.69 ± 0.37	0.68 ± 0.23	0.68 ± 0.20	0.69 ± 0.15	0.996
D1	3.97 ± 2.77	4.15 ± 2.99	3.34 ± 3.19	2.44 ± 1.83	2.02 ± 1.37	**<0.001**
D3	2.03 ± 1.32	1.50 ± 0.75	1.11 ± 0.69	0.82 ± 0.68	0.75 ± 0.40	**<0.001**

Abbreviation: PT, prothrombin time; INR, international normalized ratio; D-dimer, D-dimer; FIB, fibrin (ogen)

* One-way analysis of variance;

### Complications

Among the five groups, a total of 9 patients in the control group suffered from early wound infection or poor healing, and 1 patient in each of the other groups died. The difference among the groups was significant. No patients in any group suffered from late deep wound infection, cardiovascular or cerebrovascular events, or symptomatic VTE. The details are shown in Table 5.

**Table 5:** postoperative complications after foot and ankle fractures

	PC	LD	MD	HD	UD	*P*
Early wound infection and poor healing (< 1 month postoperative)	9	1	1	1	1	**<0.001**
Late deep infection (> 3 month postoperative)	0	0	0	0	0	1.000
Cardiovascular and cerebrovascular accident	0	0	0	0	0	1.000
Symptomatic VTE	0	0	0	0	0	1.000

Abbreviation: VTE, venous thromboembolism

## Discussion

In multiple randomized controlled clinical trials, TXA has been proven to be a safe and effective hemostatic drug that can minimize blood loss and reduce blood transfusion rates [17–20]. In joint replacement surgery, TXA significantly reduces intraoperative blood loss, the blood transfusion rate, and the periprosthetic infection rate without increasing the risk of thromboembolic events [21–23]. In arthroscopic surgery, intravenous injection of TXA reduced the postoperative drainage volume after anterior cruciate ligament reconstruction and relieved patients’ pain [24]. For orthopedic trauma, TXA can reduce hidden blood loss related to intertrochanteric fractures and tibial plateau fractures but does not increase the risk of postoperative venous thromboembolism [25–27]. In spinal surgery, TXA can significantly reduce the total perioperative blood loss volume without obvious side effects [28, 29].

However, the application of TXA in foot and ankle surgery is relatively rare [30, 31]. Currently, few studies have shown that TXA has potential benefits in reducing blood loss and wound complications without increasing the risk of thromboembolic events [32–34], but there is still some controversy [35, 36]. The foot and ankle are naturally poor in soft tissue, and soft tissue complications often occur after surgery, such as poor incision healing, incision infection or deep infection. Therefore, the application of TXA in foot and ankle fractures deserves further study.

The most important finding of this study was that, for preoperative blood loss, although there were statistically significant differences between the groups in terms of HCT on the 3rd day after admission and preoperative HBL, there was no significant difference between the HGB groups on the 3rd day after admission. Moreover, there were significant differences in HGB and HCT on the day of admission, which indicates that the general condition of patients significantly differed and had a serious impact on this section. Therefore, in this study, it was unclear whether early application of TXA could reduce preoperative HBL.

There were no significant differences in surgical blood loss or HGB or HCT levels among the five groups one day before surgery, but there were significant differences in HGB and HCT levels among the groups three days after surgery. Similarly, the HGB and HCT level variations in the HD and UD groups were lower than those in the LD and MD groups. These findings indicate that TXA can reduce surgical VBL and HBL and lower the incidence of blood transfusion and complications and that the overdose regimens have a more obvious effect than the standard-dose regimens.

TXA is an antifibrinolytic agent that binds to and inhibits plasminogen activator, thereby inhibiting the activation of plasminogen to plasmin. The main physiological role of plasmin is to degrade the fibrin network in the blood clot. In addition to fibrinolytic effects, plasmin also has proinflammatory effects. TXA can counteract all effects of plasmin. Therefore, in addition to stabilizing blood clots, TXA can also reduce the inflammatory response [37–39]. In our study, there was no significant difference in CRP levels among the five groups of patients on the day of admission. However, after TXA administration, the CRP level in the TXA group was lower than that in the control group, and the difference was more significant in the overdose groups (HD and UD groups), which shows that TXA can indeed lower the incidence of complications associated with foot and ankle fractures. In our study, there were 9 cases of incision complications in the control group and 1 case in each of the other groups; the difference among these groups was significant.

However, TXA may lead to thrombotic complications, which are the most serious problems associated with TXA. Although the results of multiple randomized controlled clinical trials have shown that the use of TXA does not increase the incidence of thrombotic events, there is also a lack of direct evidence indicating that TXA use is not directly related to complications such as DVT and pulmonary embolism (PE). In this study, the differences among the five groups in terms of preoperative and postoperative PT and INR were not significant, which shows that even when TXA is used in excess (HD and UD), it will not have a significant impact on coagulation. After administering TXA, the FIB and D-dimer levels in the TXA groups decreased, indicating that TXA had an anti-fibrinolytic effect and that the effect was more significant in the overdose groups (HD and UD). In addition, no thromboembolic events, such as VTE, occurred in the TXA group in our study, which shows that even when overdoses of TXA are used, TXA is still highly safe and will not increase the incidence of thromboembolic events.

This is the first prospective, random controlled study on whether TXA can reduce preoperative blood loss in patients with foot and ankle fractures, and this study also investigated the effectiveness and safety of intravenous application of different doses of TXA for fresh foot and ankle fractures. This study has certain limitations because the fracture styles, including pilon, ankle, and calcaneus fractures, and the fracture types and severities were diverse. In addition, the sample size of this study was small. In subsequent studies, we will increase the sample size and select a single fracture type as much as possible.

## Conclusion

On the one hand, intravenous application of TXA after foot and ankle fractures as soon as possible can reduce preoperative blood loss to the same amount as postoperative blood loss. On the other hand, TXA can also lower wound complications, and over-doses of TXA are more effective than standard doses. Moreover, overdoses of TXA do not increase the incidence of DVT.

## Data Availability

No datasets were generated or analysed during the current study.

## References

[1] Kottmeier SA, Madison RD, Divaris N (2018). Pilon fracture: preventing complications [J]. J Am Acad Orthop Surg.

[2] Heyns M, Knight P, Steve AK (2021). A single preoperative dose of Tranexamic Acid reduces Perioperative Blood loss: a Meta-analysis [J]. Ann Surg.

[3] Roberts I, Shakur H, CRASH-2 collaborators (2011). The importance of early treatment with tranexamic acid in bleeding trauma patients: an exploratory analysis of the CRASH-2 randomised controlled trial [J]. Lancet.

[4] Xiong Z, Wu K, Zhang J (2020). Different dose regimens of Intravenous Tranexamic Acid in adolescent spinal deformity surgery: a systematic review and Meta-analysis [J]. Biomed Res Int.

[5] Taeuber I, Weibel S, Herrmann E (2021). Association of Intravenous Tranexamic Acid with thromboembolic events and mortality: a systematic review, Meta-analysis, and Meta-regression [J]. JAMA Surg.

[6] Ker K, Edwards P, Perel P (2012). Effect of tranexamic acid on surgical bleeding: systematic review and cumulative meta-analysis [J]. BMJ.

[7] Thorne JG, James PD, Reid RL (2018). Heavy menstrual bleeding: is tranexamic acid a safe adjunct to combined hormonal contraception? [J]. Contraception.

[8] Weng S, Wang W, Wei Q (2019). Effect of Tranexamic Acid in patients with traumatic Brain Injury: a systematic review and Meta-analysis [J]. World Neurosurg.

[9] Brown NJ, Choi EH, Gendreau JL (2021). Association of tranexamic acid with decreased blood loss in patients undergoing laminectomy and fusion with posterior instrumentation: a systematic review and meta-analysis [J]. J Neurosurg Spine.

[10] July J, Pranata R (2020). Tranexamic acid is associated with reduced mortality, hemorrhagic expansion, and vascular occlusive events in traumatic brain injury - meta-analysis of randomized controlled trials [J]. BMC Neurol.

[11] Gong M, Liu G, Chen L (2019). The efficacy and safety of Intravenous Tranexamic Acid in reducing Surgical Blood loss in posterior lumbar Interbody Fusion for the adult: a systematic review and a Meta-analysis [J]. World Neurosurg.

[12] Hmidan Simsam M, Delorme L, Grimm D (2023). Efficacy of high dose tranexamic acid (TXA) for hemorrhage: a systematic review and meta-analysis [J]. Injury.

[13] Chornenki NLJ, Um KJ, Mendoza PA (2019). Risk of venous and arterial thrombosis in non-surgical patients receiving systemic tranexamic acid: a systematic review and meta-analysis [J]. Thromb Res.

[14] Nadler SB, Hidalgo JH, Bloch T (1962). Prediction of blood volume in normal human adults. Surg [J].

[15] Gross JB (1983). Estimating allowable blood loss: corrected for dilution [J]. Anesthesiology.

[16] Ma H, Wang H, Long X (2021). Early intravenous tranexamic acid intervention reduces post-traumatic hidden blood loss in elderly patients with intertrochanteric fracture: a randomized controlled trial [J]. J Orthop Surg Res.

[17] Charoencholvanich K, Siriwattanasakul P (2011). Tranexamic acid reduces blood loss and blood transfusion after TKA: a prospective randomized controlled trial [J]. Clin Orthop Relat Res.

[18] Kim TK, Chang CB, Kang YG (2014). Clinical value of tranexamic acid in unilateral and simultaneous bilateral TKAs under a contemporary blood-saving protocol: a randomized controlled trial [J]. Knee Surg Sports Traumatol Arthrosc.

[19] Wang CG, Sun ZH, Liu J (2015). Safety and efficacy of intra-articular tranexamic acid injection without drainage on blood loss in total knee arthroplasty: a randomized clinical trial [J]. Int J Surg.

[20] Shakur H, Roberts I, CRASH-2 trial collaborators (2010). Effects of tranexamic acid on death, vascular occlusive events, and blood transfusion in trauma patients with significant haemorrhage (CRASH-2): a randomised, placebo-controlled trial [J]. Lancet.

[21] Yang ZG, Chen WP, Wu LD (2012). Effectiveness and safety of tranexamic acid in reducing blood loss in total knee arthroplasty: a meta-analysis [J]. J Bone Joint Surg Am.

[22] Konig G, Hamlin BR, Waters JH (2013). Topical tranexamic acid reduces blood loss and transfusion rates in total hip and total knee arthroplasty [J]. J Arthroplasty.

[23] Yazdi H, Klement MR, Hammad M (2020). Tranexamic acid is Associated with reduced periprosthetic joint infection after primary total joint arthroplasty [J]. J Arthroplasty.

[24] Karaaslan F, Karaoğlu S, Yurdakul E (2015). Reducing Intra-articular Hemarthrosis after Arthroscopic Anterior Cruciate Ligament Reconstruction by the Administration of Intravenous Tranexamic Acid: a prospective, randomized controlled trial [J]. Am J Sports Med.

[25] Jiang W, Shang L (2019). Tranexamic acid can reduce blood loss in patients undergoing intertrochanteric fracture surgery: a meta-analysis [J]. Med (Baltim).

[26] Tian S, Shen Z, Liu Y (2018). The effect of tranexamic acid on hidden bleeding in older intertrochanteric fracture patients treated with PFNA [J]. Injury.

[27] Wang Z, Lu Y, Wang Q (2020). Comparison of the effectiveness and safety of intravenous and topical regimens of tranexamic acid in complex tibial plateau fracture: a retrospective study [J]. BMC Musculoskelet Disord.

[28] Lei T, Bingtao W, Zhaoqing G (2022). The efficacy and safety of intravenous tranexamic acid in patients with posterior operation of multilevel thoracic spine stenosis: a prospective randomized controlled trial [J]. BMC Musculoskelet Disord.

[29] Li J, Wang L, Bai T (2020). Combined use of intravenous and topical tranexamic acid efficiently reduces blood loss in patients aged over 60 operated with a 2-level lumbar fusion [J]. J Orthop Surg Res.

[30] Johns WL, Walley KC, Seedat R (2020). Tranexamic Acid Use in Foot and Ankle surgery [J]. Foot Ankle Orthop.

[31] Salameh M, Attia AK, El Khatib S (2022). Tranexamic acid utilization in foot and ankle surgery: a Meta-analysis [J]. Foot Ankle Int.

[32] Tang X, Li K, Zheng F (2023). The effect of perioperative tranexamic acid (TXA) in patients with calcaneal fractures: a meta-analysis and systematic review of randomized controlled trials [J]. J Orthop Surg Res.

[33] Moore AD, Smith BR, O’Leary RJ (2022). Tranexamic Acid Associated with Less Wound complications in Ankle and Hindfoot surgery: Level III, Retrospective Cohort Study [J]. J Am Acad Orthop Surg.

[34] Nodzo SR, Pavlesen S, Ritter C, et al. Tranexamic acid reduces Perioperative Blood loss and hemarthrosis in total ankle arthroplasty [J]. Am J Orthop (Belle Mead NJ). 2018;47(8). 10.12788/ajo.2018.0063. 10.12788/ajo.2018.0063 30180225

[35] Diskina BHPP, Lin D (2021). Use of tranexamic acid does not influence perioperative outcomes in ambulatory foot and ankle surgery-a prospective triple blinded randomized controlled trial [J]. Int Orthop.

[36] Steinmetz RG, Luick L, Tkach S (2020). Effect of Tranexamic Acid on Wound complications and Blood loss in total ankle arthroplasty [J]. Foot Ankle Int.

[37] Syrovets T, LuJuly O, Simmet T (2012). Plasmin as a proinflammatory cell activator [J]. J Leukoc Biol.

[38] Prudovsky I, Kacer D, Zucco VV (2022). Tranexamic acid: beyond antifibrinolysis [J]. Transfusion.

[39] Lei YT, Xie JW, Huang Q (2020). The antifibrinolytic and anti-inflammatory effects of a high initial-dose tranexamic acid in total knee arthroplasty: a randomized controlled trial [J]. Int Orthop.

